# The impact of rare and low-frequency genetic variants in common disease

**DOI:** 10.1186/s13059-017-1212-4

**Published:** 2017-04-27

**Authors:** Lorenzo Bomba, Klaudia Walter, Nicole Soranzo

**Affiliations:** 1 Human Genetics, Wellcome Trust Sanger Institute, Genome Campus, Hinxton, CB10 1HH UK; 2 Department of Haematology, University of Cambridge, Hills Rd, Cambridge, CB2 0AH UK; 3 The National Institute for Health Research Blood and Transplant Unit (NIHR BTRU) in Donor Health and Genomics at the University of Cambridge, University of Cambridge, Strangeways Research Laboratory, Wort’s Causeway, Cambridge, CB1 8RN UK

## Abstract

Despite thousands of genetic loci identified to date, a large proportion of genetic variation predisposing to complex disease and traits remains unaccounted for. Advances in sequencing technology enable focused explorations on the contribution of low-frequency and rare variants to human traits. Here we review experimental approaches and current knowledge on the contribution of these genetic variants in complex disease and discuss challenges and opportunities for personalised medicine.

**Electronic supplementary material:**

The online version of this article (doi:10.1186/s13059-017-1212-4) contains supplementary material, which is available to authorized users.

## Introduction

Genetic research has played an instrumental role in the discovery of new biological pathways underpinning complex human disease and the evaluation of new targets for therapeutic development. The past decade has seen an exponential increase in the number of known genetic loci predisposing to complex disease, enabled by large-scale meta-analyses based on genome-wide single-nucleotide polymorphism (SNP) arrays imputed into reference haplotype panels [1]. These efforts have identified thousands of (mostly common) genetic loci associated with disease biomarkers and disease endpoints [2], with some initial examples of how these genetic findings can be used to inform disease prediction [3], identification of causal mechanisms of disease [4, 5] and the prioritisation of new biological targets in drug discovery programmes [6–8].

Many challenges continue to exist in both the discovery and interpretation of findings from genome-wide association studies (GWASs). Highly successful international collaborative efforts have enabled association studies to reach unprecedented sizes of thousands to hundreds of thousands of study participants [9–12]. Despite the increases in statistical power afforded by these large-scale studies, for the majority of human traits genetic associations discovered account for a fraction of disease or trait heritability (the “missing heritability” paradigm). Genetic variants that are outside the reach of the most statistically powered association studies [13] are thought to contribute to the missing heritability of many human traits, including common variants (here denoted by minor allele frequency [MAF] >5%) of very weak effect, low-frequency (MAF 1–5%) and rare variants (MAF <1%) of small to modest effect, or a combination of both, with several possible scenarios all deemed plausible in simulation studies [14].

Empirical studies attempting to understand the impact of rare or less common variation on human complex diseases and traits remain to date relatively limited [15, 16], but some lessons on their properties are beginning to emerge from exome-wide and genome-wide sequencing studies. For most traits, these studies have demonstrated an inverse relationship between the variant’s “regression effect size” (or disease odds ratio) and its frequency in the population, as predicted by population genetic models [17]. Differential selective pressures acting on variants across the allele frequency spectrum underpin the observed shape of this relationship in different human traits. Such a relationship tends to be skewed in favour of rare variants for traits most strongly influenced by natural selection, compared with quantitative phenotypes or late-onset diseases [17]. Mendelian diseases are at the extreme end of the spectrum because of the high impact of selection on transmission of rare variants to subsequent generations. Initial evidence for complex diseases suggests that autism spectrum disorders may be skewed towards rarer susceptibility variants [18] compared with diseases such as type 2 diabetes [19], age-related macular degeneration [15] and schizophrenia [20], and quantitative cardiometabolic traits [21, 22]. Further efforts to discover associations driven by low-frequency and rare variants through genome sequencing and large-scale imputation efforts allow continuous refinements of the proportion of trait heritability explained by variants across the frequency spectrum [23]. Finally, it is worth noting that estimates of missing heritability from genome-wide variants are strongly dependent on assumptions on linkage disequilibrium, allele frequency and genotype certainty [13, 24]. Rare SNPs have been estimated to contribute substantial fractions of heritability (half the heritability of common SNPs [25]), but these early estimates will likely be revised as data continue to be accrued.

Another important challenge for complex disease genetics is the identification and functional characterisation of causal variants, or mutations in relevant genes, responsible for association signals detected through GWASs [26]. Common risk variants map overwhelmingly to regulatory regions [12], where inference of the underlying causative genes is difficult. Recent developments in cellular and functional genomics provide effective strategies to annotate the clinical and phenotypic consequences of genome sequence variation [27]. These approaches, which investigate a range of processes such as transcription, translation and epigenetic regulation at the organismal, physiological or cellular level [28], are a necessary step towards our understanding of the complex relationship between genotype and phenotype on a global (genome-wide) scale. Even in the presence of expansive datasets for annotation, however, the interpretation of the precise functional consequence of each variant requires rigorous and often painstaking evaluation of many genes in different possible cellular and environmental contexts [29]. On the other hand, rare variants in or near gene targets display larger average effects on phenotype compared with both regulatory variants of comparable allele frequencies and common genetic variants [21, 30]. The discovery of these variants through focused sequencing explorations of protein-coding regions is expected to greatly facilitate the task of annotating genes underpinning genetic associations with complex disease and describing the functional consequences of human sequence variation. There are, therefore, compelling arguments to accelerate efforts to identify variants within these regions because of the relative ease with which these discoveries can be turned into biological insights.

Here we review the current state of knowledge from rare variant association studies (RVASs) of complex traits and review approaches for discovering and testing associations for rare variants. Further, we discuss the growing body of literature documenting examples of highly clinically informative genetic variants identified through bespoke genotyping arrays, imputation and population-scale whole-exome and whole-genome sequencing.

## Genomic tools for assessing low-frequency and rare variants

Three broad strategies are available to access low-frequency and rare variants: genotype imputation, the use of custom genotyping arrays and the use of whole-exome or whole-genome sequencing.

### Imputation

Genotype imputation provides a cost-effective strategy for expanding the SNP content of genome-wide genotyping arrays. It relies on the availability of reference panels of phased haplotypes that can be used to impute genotypes into sparse datasets generated by commercial genotyping arrays [31, 32]. Multiple different reference panels have been generated since 2005, enabled by expanding collections of polymorphisms in human populations. The first two widely used reference panels generated by the HapMap project included 269 samples and just over one million SNPs (phase I) [33] and 3.1 million SNPs (phase II) [34], respectively. The ascertainment of these early panels was strongly skewed towards common variants (MAF >5%) found near human genes, thus limiting the representation of low-frequency and rare variants in early GWASs [35]. HapMap phase III included 1.6 million SNPs in 1184 individuals from 11 populations, ascertained by common SNP repositories and from targeted resequencing of ten 100-kb regions in 692 of these individuals. Compared with previous reference panels, the authors demonstrated gains in imputation accuracy particularly for low-frequency and rare variants [36].

Further improvements in imputation panels were enabled by large-scale whole-genome sequencing (WGS) efforts in reference human populations, and particularly the 1000 Genomes Project (pilot, phase I and phase III). In the first phase of the project (phase I), a combination of low read depth WGS (2–4×) and targeted deep (50–100×) exome sequencing was used to characterise 38 million single-nucleotide variants (SNVs) and 1.4 million short insertion-deletions (INDELs) in 1092 individuals from 14 populations. The authors further showed that individuals from the various populations display different profiles of rare and common variants with considerable geographic differentiation [37]. The data set was expanded in phase III where the genomes of 2504 individuals from 26 populations were reconstructed by applying a combination of low-read-depth WGS, deep exome sequencing and dense microarray genotyping. This resulted in over 88 million variants which were phased onto high-quality haplotypes. The authors estimated that this resource includes **>**99% of SNVs with a frequency of **>**1% [38].

In addition to the 1000 Genomes Project, which comprises samples from all over the world, other panels based on WGS have been generated in individual populations. One of these efforts was the UK10K Cohorts Project, which carried out low-read-depth (approximately 7×) WGS in 3781 individuals of British ancestry from two population-based cohorts. Overall, the project identified over 42 million SNVs and 3.5 million INDELs, of which about 80% were rare and about 5% were low frequency, and in total 24 million were novel variants. The UK10K WGS imputation reference panel was shown to increase coverage and accuracy in European populations, especially for low-frequency and rare variants, when compared with the 1000 Genomes Project phase I (1000GP) reference panel (where the European sample comprises only about 10% of the UK10K sample size) [39]. Zheng and co-authors demonstrated the value of using a combined UK10K/1000 Genomes Project reference panel to discover low-frequency variants associated with bone mineral density [40]. Other sequencing studies, such as Genome of the Netherlands (GoNL) [41], SardiNIA [42, 43] and HELIC-MANOLIS [44], also reported the usefulness of population-specific samples for the characterisation of rare variants.

Finally, efforts are now in place to combine publicly available WGS datasets to create a single reference panel with increased depth of low-frequency and rare haplotypes. To date, the Haplotype Reference Consortium has combined low-read-depth WGS data (4–8×) from 20 studies of mainly European ancestry. The relative panel contains 64,976 haplotypes from 39,235,157 SNVs with minor allele count ≥5, and the large number of samples and variant sites increases the accuracy of the genotype imputation, especially at low-frequency variants down to 0.1% MAF and allows efficient phasing and imputation on existing servers with the aim to carry out imputation in a more streamlined manner [45, 46]. The Haplotype Reference Consortium panel will continue to incorporate samples from worldwide populations, which is important; since rare variants are, on average, younger than common variants, they show more geographical clustering and they are more difficult to impute. In order to provide a comprehensive imputation reference panel, it is important to combine many samples and to include samples from the geographical area of interest [47]. Additional advances to current reference panels are likely to emerge from large-scale sequencing studies such as the Trans-Omics for Precision Medicine (TOPMed) Program [48] or the 100,000 Genomes Project in the UK [49].

### Custom genotyping arrays

An alternative strategy to imputation to survey low-frequency and rare variants in association studies takes advantage of bespoke genotyping arrays. These arrays are often disease focused and aim to enrich standard haplotype tagging SNP panels with variants of interest identified through sequencing and fine-mapping efforts. One such array was Immunochip, designed in 2009 by investigators of 11 distinct autoimmune and inflammatory diseases to assay 195,806 SNPs and 718 small INDELs. It included the top 2000 independent variants for each disease that showed evidence for an association, as well as SNPs from the 1000 Genomes Project and resequencing data to densely cover 186 different disease loci, including the major histocompatibility complex (MHC) and the killer immunoglobulin-like receptor (KIR) loci. The coverage of the low-frequency and rare variant spectrum is incomplete since the array was designed using early 1000 Genomes Pilot data (February 2010 release). Another limitation of the Immunochip is that the design is based on studies of European samples, and thus non-European variation is under-represented in this array [50].

The Metabochip custom array interrogates nearly 200,000 SNP markers of 257 genome-wide significant association signals for metabolic diseases (type 2 diabetes, coronary artery disease, myocardial infarction) and quantitative traits (body mass index, glucose and insulin levels, lipid levels and blood pressure). This array, similar to Immunochip, was very cost-effective, meaning more samples could be genotyped and its uniformity enabled direct comparison across phenotypes [51]. Metabochip SNPs were selected from International HapMap [34] and 1000 Genomes Projects [52] repositories to include SNPs across a wide range of allele frequencies. Metabochip SNPs focus on trait-associated loci (1.5% of the genome) by increasing their SNP resolution by fine-mapping. Imputation accuracy in fine-mapping regions is increased compared to traditional SNP arrays, as 54.4% of European SNPs from 1000GP phase I are tagged with *r* ^2^ ≥ 0.8 [51].

More recently, custom genotyping arrays have been developed to enhance representation of low-frequency and rare variants genome-wide. The UK Biobank Axiom Array contains 820,967 genetic variants, targeting specifically disease-specific and rare coding variants [53]. The Illumina HumanExome BeadChip (ExomeChip) comprises 247,870 variants (of which about 75% have MAF **<**0.5%) discovered through exome sequencing in approximately 12,000 individuals, including high-confidence non-synonymous and protein-altering variants (splice-site and stop gain or loss codons). Additionally, the exome chip includes common variants found through GWAS, ancestry informative markers (for African and Native Americans), mitochondrial variants, randomly selected synonymous variants, HLA tag variants and Y chromosome variants. The widespread application of the ExomeChip array has resulted in relatively few novel discoveries, including the identification of novel associations of a low-frequency coding variant in *GLP1R* with fasting glucose and type 2 diabetes [54], a number of novel low-frequency lipid signals at previously known loci [55, 56] and a large set of 32 rare and 51 low-frequency coding variants associated with height [57].

### Exome or whole-genome sequencing

Historically, candidate gene sequencing studies have been used to explore sequence variation through relatively small-scale sequencing efforts. These were based mainly on capillary (Sanger) sequencing, typically focused on small numbers of patients and healthy controls and on genes with a strong a priori biological candidacy or importance for a given trait of disease [58–64]. Studies based on whole-exome sequencing (WES) and WGS have been increasingly used to systematically assess the properties and associations of rare variants, enabled by decreases in sequencing costs and increases in sequencing throughput [65]. WES probes only approximately 1.2% of the genome, and is thus cheaper relative to WGS, but limits investigations to variants in protein-coding regions of the genome. An enrichment analysis in the UK10K Project used functional and regulatory features, such as genic annotations, chromatin states, DNaseI hypersensitive sites, transcription factor binding sites, conservation scores and histone modifications, to assess the relative contribution of low-frequency and common variants to associations. The results showed that low-frequency variants in exonic regions displayed the strongest degree of enrichment (25-fold, compared with fivefold for common variants), which is compatible with the signatures of purifying selection, such as a negative correlation between functionally important variants and allele frequency [66]. However, non-coding low-frequency alleles were shown to also contribute to phenotypic trait variation: both common and low-frequency variants had comparably strong levels of functional enrichment for several non-coding domains (i.e. transcription start sites, DNase I hotspots and 3′ UTRs of genes) [21]. Additionally, it has been suggested that the quality and the calling of coding SNVs and INDELs is comparable if not better in WGS, i.e. an estimated 3% of coding variants were found by WGS but not called by WES [67]. We review later results of recent exome- and genome-sequencing studies of complex disease.

## Optimal methods for association analysis with low-frequency and rare variants

Approaches typically used for testing associations of genetic variants with phenotype based on simple regression models are underpowered for rare variants [68]. Moreover, many more rare independent variants are found throughout the genome compared with common variants, increasing the multiple testing penalty for these studies. To overcome both of these issues, several statistical methods have been proposed to increase statistical power in association studies, typically by seeking to combine information across multiple rare variants within a specific genomic functional unit (e.g. gene, exon). Rare variant region-based methods can be grouped in four broad categories (Table 1).

**Table 1:** Summary of the features, the pros and cons of the different type of methods described in this review and the software currently available

Type of method	Methods	Main features	Ability to discriminate risk and protective alleles	Range of study designs they can be applied to	Allelic architecture scenarios the method is compatible with	Available software
Burden test	ARIEL test [53], RWAS [54], CAST [55], CMC method [56], MZ Test [57], WSS [58], aSum [59], Step-up [60], EREC test [61], VT [62], KBAC method [63], RBT [64]	Collapsing genetic variants into a single score, assumption that tested variants are all causal and associated with the trait with the same direction and magnitude of effect	No	Causal variants, e.g. loss of function (LoF) variants	All variants have the same direction and magnitude of effect	ARIEL, EPACTS, GRANVIL, PLINK/SEQ, Rvtests, SCORE-Seq, SKAT, VAT, KBAC, RAREMETAL
Variance-component test	C-Alpha test [67], SKAT [68], SSU test [69], KBAT [70]	Allowing for both risk and protective alleles, i.e. tested variants can have different directions of effect	Yes	Applicable to all available variants, possibly using some weighting strategy	Variants can have opposing directions of effect	EPACTS, PLINK/SEQ, SCORE-Seq, SKAT, VAT, RAREMETAL
Combined test	SKAT-O [71], EMMPAT [72], Fisher method [73], MiST [74]	Combining results from two or more complementary tests	Yes	Applicable to all available variants, possibly using some weighting strategy	Variants can have both opposing or same direction of effect	EPACTS, PLINK/SEQ, MiST, SKAT, RAREMETAL
Other tests	LASSO [75], EC [76]	Accounting for signal sparsity	No	Applicable to all available variants, possibly using some weighting strategy	Variants are sparse	MENDEL

*ARIEL* accumulation of rare variants integrated and extended locus-specific, *aSum* data-adaptive sum test, *CAST* cohort allelic sums test, *CMC* combined multivariate and collapsing, *EC* exponential combination, *EPACTS* efficient and parallelisable association container toolbox, *EREC* estimated regression coefficient, *GRANVIL* gene- or region-based analysis of variants of intermediate and low-frequency, *KBAC* kernel-based adaptive cluster, *MiST* mixed-effects score test for continuous outcomes, *MZ* Morris and Zeggini, *RBT* replication-based test, *Rvtests* rare-variant tests, *SKAT* sequence kernel association test, *SSU* sum of squared score, *VAT* variant association tools, *VT* variable threshold, *WSS* weighted-sum statistic

### Burden tests

Burden tests (ARIEL test [69], RWAS [70], CAST [71], CMC method [72], MZ Test [73], WSS [74], aSum [75], Step-up [76], EREC test [77], VT [78], KBAC method [79], RBT [80]) collapse information for genetic variants within a predefined functional unit into a single score and then regress this score against the trait of interest. The various burden tests differ in how this information is summarised. For example, the simplest form of burden test counts the number of minor alleles across all variants in the set producing a genetic score for each individual [69]. The cohort allelic sums test (CAST) [71] sets the genetics score to 0 or 1 based on the presence or absence, respectively, of at least one rare variant in the region tested. A more sophisticated weighting function was proposed by Madsen and Browning [74] with the weighted sum statistic (WSS) that takes into account all the variants’ frequencies without the need to set a fixed threshold to define rare and common variant as in CAST. Moreover, WSS considers other information on functional annotation of variants in its weighting method. Other kinds of burden tests have been developed to combine the collapsing methods with a multivariate test, such as the combined multivariate and collapsing (CMC) method [72]. Main limitations of burden tests are the strong assumption that the variants tested within the functional unit are all causal and associated with the trait with the same direction and magnitude of effect. This assumption is violated most of the time due to the highly variable and unknown allelic architecture of complex traits. For example, the *PCSK9* gene carries alleles with both loss and gain function effects on LDL cholesterol [81, 82].

### Variance-component tests

Varience-component tests (C-Alpha test [83], SKAT [84], SSU test [85], KBAT [86]) have been developed to consider the particular scenario where both risk and protective alleles may be found within a given gene or functional unit, testing for the distributions of genetic effects within a set of variants. This approach is flexible and allows for a mixture of effects in the rare variant set. The sequence kernel association test (SKAT) is one of the most widely used approaches, can take into account weightings of rare variants, family structure and covariates and is primarily designed for quantitative traits. Other tests (C-alpha [a special case of SKAT], WSS and CMC) can be applied only in case–control studies [84].

### Combined tests

Combined tests (SKAT-O [87], EMMPAT [88], Fisher method [89], MiST [90]) have been developed to maximise power in a broad range of allelic architecture scenarios. In fact, this is the more realistic assumption and there are a number of statistical approaches to combine *p* values from two or more complementary tests. Among these approaches Fisher’s method [89] has been extensively used. More recently Lee and colleagues proposed an optimisation of the SKAT test (SKAT-O) that combines the burden and SKAT tests considering their best linear combination [87, 91].

### Other tests

Other tests have been developed to account for signal sparsity across the tested region and include least absolute shrinkage and selection operator (LASSO) and the exponential combination (EC) test [92, 93]. Also Bayesian approaches have been proposed, but due to the computational time they are not as widely used as the aforementioned frequentist approaches [94]. A critical problem is to account for sequence quality, especially in next-generation sequencing data with relatively low coverage per individual. Two previous approaches are able to incorporate weights based on genotype uncertainty metrics for imputed genetic variants or for sequencing-derived variants [95], outperforming some pre-existing models [96].

## Power, replication and confounding affecting rare variant association tests

An ongoing challenge is to systematically evaluate the relative merit, assumptions, implementation and statistical power of different analyses. Attempts to systematically evaluate the power of different methods for different allelic predisposition scenarios have been carried out using both simulations and empirical data [68, 69, 97–99]. They have shown that gene-based tests are sensitive to variables such as the choice of analysis unit (e.g. exon versus whole gene), the number of variants tested within an aggregation unit and also the choice of particular functional classes of variants (e.g. loss-of-function, non-synonymous, etc.) or the magnitude of linkage disequilibrium between variants. As an example, Moutsianas and colleagues carried out a comprehensive study based on simulated data of similar size to current next-generation sequencing (NGS)-based association studies (3000 case–control individuals) [68]. The authors assessed power to detect associations using the main gene-based rare variant tests and for six different architecture scenarios informed by an empirical study of type 2 diabetes (T2D) (described in [68]). They showed that power to discover associations was low (<20%, for type I error (α) = 2.5 × 10^–6^), and even with sample sizes more than triple those of current empirical studies (about 10,000 case–control individuals) the power remained modest (on average about 60%). The authors further showed that combined tests (e.g. SKAT-O and MiST) had marginally greater power to detect associations across the number of simulated allelic architectures. This suggests that the application of these tests may be preferable in the context of genome-wide explorations in order to capture the widest possible range of allelic scenarios at different genes. Burden tests were shown to have more power to identify associations for deleterious variants, especially when neutral variation is filtered out. However, it is still unclear to what extent the simulations used in this and other studies may reflect the true allelic architecture of traits, highlighting the importance of implementing flexible testing scenarios in RVASs.

Other strategies for increasing statistical power are also liable to potential problems. For instance, the benefits of increases in sample size that are achieved through combining different sequencing studies can potentially be outweighed by issues of heterogeneity in disease state or in environmental exposures, or even differences in allele frequency between studies. Furthermore, studies focusing solely on certain categories of variants (e.g. loss of function variants) could on one hand increase the power by only considering variants with strong effect on phenotype. On the other hand, it has been suggested that removing flanking variants could potentially decrease the overall power to detect an association signal [100]. To address these issues, Liu et al. [101] developed a new method to meta-analyse rare variants that instead of using *p* values combines score statistics for each individual variant and employs a covariance matrix between variants reflecting the linkage disequilibrium structure inside the tested region.

Another challenge for RVASs is to achieve robust replication of signals, particularly in the instances where associations present allelic and locus heterogeneity [102]. For rare variants identified through single variant association tests, replication can be achieved by genotyping the identified variant in replication cohorts, provided obviously that the variant is indeed polymorphic in that cohort. For variants identified through aggregation methods, replication may be achieved by genotyping all the variants within the functional units discovered or direct sequencing of all the functional units [103]. Advances in sequencing and target-capture technologies reduce the cost of resequencing and, although it is more expensive than genotyping, resequencing can potentially identify new variants inside the functional unit that the discovery cohorts were not able to pinpoint [104, 105].

Finally, population stratification poses unique challenges in RVASs. In fact, systematic differences in allele frequencies due to differences in ancestry are more pronounced for rare variants [37]. Moreover, strong patterns of population stratification are predicted to arise in the presence of sharp spatial distributions for non-genetic risk of disease [106]. Adjusting for population stratification using traditional methods such as principal component analysis (PCA) and linear mixed effect models may, in most of the cases, not be suitable for rare variant tests [106–109]. Alternatives to reduce the confounding effects of population stratification in rare variant tests are using family-based designs or including spatial/geographical information [21, 106]. Moreover, calculating principal components using all or only common variants has shown to be more effective than using only rare variants [110]. Babron et al. [111] reported differences in population stratification patterns between rare and common variants in the UK population.

## Study designs for enriching or prioritising rare variants

Study designs exploiting unique characteristics of different populations have been used to boost power in association studies of rare and low-frequency alleles. One notable example is population isolates, which provide powerful study designs for medical genetics due to a number of advantageous characteristics. For example, variants of medical importance that are rare in outbred populations might be found at higher frequencies in isolated populations due to past bottleneck events, genetic drift or adaptation and selection [43, 112], increasing power to detect associations with medically important phenotypes [113, 114].

A particularly interesting case of rare variation is variants that lead to inactivation of the corresponding protein. Such so-called loss-of-function (LoF) variants include variants predicted to lead to premature termination of the protein (stop-gain variants or protein-truncating variants) and insertion or deletion polymorphisms that affect the overall codon sequence of the protein (frameshift INDELS) or alter pre-mRNA splicing of essential exons (essential splice-site variants). LoF variants provide powerful tools to understand the impact of “knocking out” human genes, akin to gene knockout experiments commonly conducted in model organisms [115]. Understanding the phenotypic and clinical consequences of carrying LoF alleles, particularly when they are carried in the homozygous (i.e. complete knockout) state, has been shown to provide crucial insights into the identification of new disease genes and druggable pathways [116–118]. Further, studies of LoF variants in established drug targets, when carried by an otherwise healthy individual, provide evidence for safety of modulating that particular target to reduce disease risk. The data set of 60,706 individuals collated by the Exome Aggregation Consortium (ExAC) can assist in filtering of candidate disease-causing variants and in the discovery of human “knockout” variants in protein-coding genes [119].

Efforts to discover these mutations are boosted in populations with high rates of homozygosity, for example in populations with a tradition of consanguineous marriage, and where such variants occur more often in a homozygous state. Analysing samples from the PROMIS study, it was found that 961 genes were completely inactivated in at least one participant. Combined with rich phenotype information, this enabled the discovery of genotype–phenotype associations of clinical importance, such as the association of *APOC3* with absent plasma apolipoprotein C-III levels [120]. Another study predicted LoF in 781 genes after analysing 3222 British Pakistani heritage adults with high parental relatedness [121]. The whole genomes of 2636 Icelanders together with imputing additional 101,584 chip-genotyped and phased Icelanders has begun to enable studies of rare complete human gene knockouts in the Icelandic population. The authors are also planning to characterise most homozygous LoF variants in the Icelandic population and to carry out bespoke phenotyping of the carriers [122]. A caveat of this approach is that the functional consequences of sequence variants are typically bioinformatically annotated as based on generic transcript annotations (for instance based on the most deleterious consequence among all annotated transcripts). LoF variants may therefore not lead to protein inactivation in a biologically relevant context, which could be due to gene redundancy, or to heterozygosity, or to genuine variants that do not actually disrupt gene function, or to variants that are only active in certain tissue-specific (or rare) isoforms [112, 115]. Thus, extensive and painstaking follow-up efforts are required to validate the predicted consequences of these variants.

## Initial results from associations from large-scale sequencing projects

**Fig. 1:**
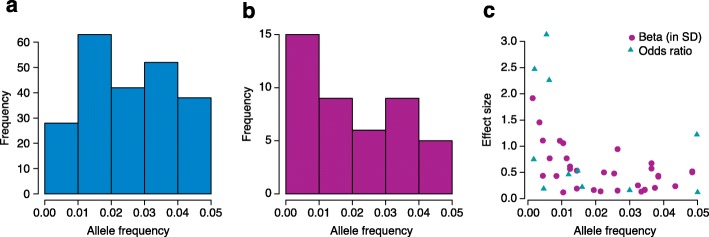
The allele frequency spectrum for **a** genome-wide association study variants (Additional file 1) and **b** sequenced variants that were associated with a variety of traits (Table 3 and Additional file 1). There is a clear shift to lower allele frequencies for variants discovered in sequencing studies. **c** The effect size versus allele frequency for sequenced variants; i.e. to detect associations that involve variants with lower allele frequencies, higher effect sizes are needed or large sample sizes. Effect size is usually measured as “beta” for quantitative traits and as “odds ratio” for dichotomous traits

A growing number of studies have explored properties of low-frequency and rare variants and their relevance for complex traits and disease (Fig. 1, Tables 2, 3, Additional file 1). A first exploration based on exome-sequencing in 200 individuals from Denmark identified an excess of low-frequency deleterious, non-synonymous SNVs compared with synonymous SNVs [123]. In another study 15,585 human protein-coding genes were sequenced to an average median depth of 111× in 2440 individuals of European and African ancestry. The majority of the SNVs were rare (MAF <0.5%), previously unknown and population-specific. It was estimated that 2.3% of the 13,595 SNVs each person carried were predicted to affect protein function of about 313 genes per genome and most of the variants that affected the protein function were rare [66].

**Table 2:** Overview of the sequencing studies

Cohort	Number of samples	Type (WGS or WES)	Coverage	Population	Disease/traits	Source
UK10K	3715	WGS	6.5×	UK	Across diseases	http://www.uk10k.org/
Sardinia	3445	WGS	4×	Sardinian	Lipids	https://sardinia.irp.nia.nih.gov/
IBD	4478	WGS	4 × + 2×	UK	Inflammatory bowel disease	http://www.ibdresearch.co.uk/
GoT2D	2710	WGS/WES	4×/Exome	UK	Type 2 diabetes	www.type2diabetesgenetics.org
BRIDGES	2487	WGS	6–8× (12×)	European	Breast cancer	https://bridges-research.eu/
1000 Genomes	2495	WGS/WES	4×/Exome	Multiple populations	–	http://www.1000genomes.org/
GoNL	748	WGS	12×	Dutch	Cardiovascular disease	http://www.nlgenome.nl/
AMD	3305	WGS	4×	European, Asian	Age-related macular degeneration	http://eaglep.case.edu/iamdgc_web/
HUNT	1023	WGS	4×	Norwegian	Across diseases	https://www.ntnu.edu/hunt
SiSu + Kuusamo	1918	WGS	4×	Finnish	Cardiovascular disease	http://www.sisuproject.fi/
INGI-FVG	250	WGS	4–10×	Italian	Across diseases	http://www.netgene.it/ita/ingi.asp
INGI-Val Borbera	225	WGS	6×	Italian	Across diseases	http://www.netgene.it/ita/ingi.asp
INGI-Carlantino	94	WGS	4×	Italian	Across diseases	http://www.netgene.it/ita/ingi.asp
MCTFR	1325	WGS	10×	–	Developmental disease	https://mctfr.psych.umn.edu/
HELIC	247	WGS	4× (1×)	Greek isolates	Across diseases	http://www.helic.org/
ORCADES	398	WGS	4×	Orkney islands	Across diseases	http://www.orcades.ed.ac.uk/orcades/
InCHIANTI	676	WGS	7×	Tuscan	Aging	http://inchiantistudy.net
GECCO	1131	WGS	4–6×	Across populations	Colorectal cancer	https://www.fredhutch.org/en/labs/phs/projects/cancerprevention/projects/gecco.html
GPC	697	WGS	30×	Dutch	Personality traits	http://www.tweelingenregister.org/GPC/
Project MinE	935	WGS	45×	Across populations	Amyotrophic lateral sclerosis	http://projectmine.com
NEPTUNE	403	WGS	4×	Across populations	Nephrotic syndrome	http://www.neptune-study.org/
deCODE	2636	WGS	20×	Icelandic	Across diseases	http://www.decode.com/
CHARGE	962	WGS	6×	Across populations	Cardiovascular disease, ageing	http://www.chargeconsortium.com/
ESP of NHLBI	6500	WES	Up to 111×	Across populations	Across diseases	https://esp.gs.washington.edu/drupal/
T2D-GENES	12,940	WES	82×	Across populations	Type 2 diabetes	http://www.type2diabetesgenetics.org/projects/t2dGenes

*ESP* Exome Sequencing Project, *NHLBI* National Heart, Lung, and Blood Institute, *WES* whole-exome sequencing, *WGS* whole-genome sequencing

**Table 3:** Rare variants (AF <5%) discovered in WGS, WES and imputed studies and found to be associated with various traits

Gene	Variant ID	Trait/disease	Samples	AF (cases/controls)	Beta/OR (SE)/(CI)	Type	Study	Population	Reference
*PCSK9*	rs11591147	LDL cholesterol	3621	0.022	–0.470 (0.085)	WGS	UK10K	British	http://www.ncbi.nlm.nih.gov/pubmed/26367797
*PCSK9*	rs11591147	LDL cholesterol	6602	0.038	–0.406 (0.053)	WGS + imputation	SardiNIA	Sardinian	http://www.ncbi.nlm.nih.gov/pubmed/26366554
*PCSK9*	rs11591147	Triglycerides	6602	0.038	–0.390 (0.053)	WGS + imputation	SardiNIA	Sardinian	http://www.ncbi.nlm.nih.gov/pubmed/26366554
*EN1*	rs55983207	BMD	45,436	0.05	0.12	WGS + WES + imputation	UK10K and others	European	http://www.ncbi.nlm.nih.gov/pubmed/26367794
*EN1*	rs11692564	BMD	40,516	0.016	0.22	WGS + WES + imputation	UK10K and others	European	http://www.ncbi.nlm.nih.gov/pubmed/26367794
*EN1*	rs188303909	BMD	40,453	0.03	0.16	WGS + WES + imputation	UK10K and others	European	http://www.ncbi.nlm.nih.gov/pubmed/26367794
*CCDC36*	rs202238847	Height	51,309	0.021	0.1091 (0.0233)	WGS + imputation	UK10K	British	Tachmazidou et al. (in press)
*ADIPOQ*	rs74577862	Adiponectin	3621	0.026	–0.915 (0.091)	WGS	UK10K	British	http://www.ncbi.nlm.nih.gov/pubmed/26367797
*ADIPOQ*	rs17366653	Adiponectin	3621	0.01	–1.029 (0.150)	WGS	UK10K	British	http://www.ncbi.nlm.nih.gov/pubmed/26367797
*GHR*	rs121909358	Height	6307	0.0087 (<0.0001)	–0.64	WGS + imputation	SardiNIA	Sardinian	http://www.ncbi.nlm.nih.gov/pubmed/26366551
*TMEM161B*	rs774396010	HDL cholesterol	3621	0.001	–1.887 (0.378)	WGS	UK10K	British	http://www.ncbi.nlm.nih.gov/pubmed/26367797
*PAM*	rs35658696	Type 2 diabetes	278,254	0.0498	1.22	WGS + imputation	deCODE	Icelandic	http://www.ncbi.nlm.nih.gov/pubmed/24464100
*GLP1R*	rs10305492	Fasting glucose	60,564	0.01	–0.09 (0.013)	GWAS + WES	CHARGE	African and European	http://www.ncbi.nlm.nih.gov/pubmed/25631608
*TREM2*	rs75932628	Alzheimer’s disease	110,050	0.0063	2.26	WGS + imputation	deCODE	Icelandic	http://www.ncbi.nlm.nih.gov/pubmed/23150908
*CCND3*	rs112233623	MCV	107,686	0.011	0.739 (0.05)	WGS + imputation	UK10K and others	European	https://www.ncbi.nlm.nih.gov/pubmed/27668658
*WNT16*	rs148771817	BMD	10,387	0.012	0.46	WGS + WES + imputation	UK10K and others	European	http://www.ncbi.nlm.nih.gov/pubmed/26367794
*ABCA1*	rs3824477	HDL cholesterol	56,598	0.026	0.123 (0.016)	WGS + imputation	UK10K and others	European	https://www.ncbi.nlm.nih.gov/pubmed/27668658
*FNBP1*	rs528899443	FEV1/FVC	3621	0.004	1.078 (0.204)	WGS	UK10K	British	http://www.ncbi.nlm.nih.gov/pubmed/26367797
*GFI1B*	rs150813342	PLT	114,753	0.004	–0.406 (0.026)	WGS + imputation	UK10K and others	European	https://www.ncbi.nlm.nih.gov/pubmed/27668658
*GFI1B*	rs150813342	PLT	13,744	0.008	–0.402 (0.07)	WES	Six cohort studies	European and African American	http://www.ncbi.nlm.nih.gov/pubmed/27486782
*ANK3*	rs141471070	FEV1/FVC	3621	0.006	0.739 (0.164)	WGS	UK10K	British	http://www.ncbi.nlm.nih.gov/pubmed/26367797
*KCNQ1*	rs150199504	Height	6307	0.07 (<0.01)	–0.31	WGS + imputation	SardiNIA	Sardinian	http://www.ncbi.nlm.nih.gov/pubmed/26366551
*HBB*	rs11549407	Cholesterol, total	6602	0.048	–0.490 (0.05)	WGS + imputation	SardiNIA	Sardinian	http://www.ncbi.nlm.nih.gov/pubmed/26366554
*HBB*	rs11549407	LDL cholesterol	6602	0.048	–0.473 (0.051)	WGS + imputation	SardiNIA	Sardinian	http://www.ncbi.nlm.nih.gov/pubmed/26366554
*SMCO4*	rs111902751	FVC	3621	0.014	–0.505 (0.103)	WGS	UK10K	British	http://www.ncbi.nlm.nih.gov/pubmed/26367797
*APOA5*	rs778114184	Triglycerides	6602	0.025	–0.450 (0.064)	WGS + imputation	SardiNIA	Sardinian	http://www.ncbi.nlm.nih.gov/pubmed/26366554
*APOC3*	rs138326449	Triglycerides	3621	0.003	–1.425 (0.265)	WGS	UK10K	British	dx.doi.org/10.1038/ncomms5871
*APOC3*	rs138326449	Triglycerides	3734	0.001	NA	WES	ESP of NHLBI	European or African	http://www.ncbi.nlm.nih.gov/pubmed/24941081
*APOC3*	rs138326449	VLDL	3621	0.003	–1.426 (0.265)	WGS	UK10K	British	dx.doi.org/10.1038/ncomms5871
*CCND2*	rs76895963	Type 2 diabetes	278,254	0.0147	0.53	WGS + imputation	deCODE	Icelandic	http://www.ncbi.nlm.nih.gov/pubmed/24464100
*VWF*	rs61750625	VWF antigen	4468	0.00763	–39.6 (9.71)	WES	ESP of NHLBI	European or African	http://www.ncbi.nlm.nih.gov/pubmed/23690449
*VWF*	rs149424724	VWF antigen	4468	0.008	–40.3 (10.0)	WES	ESP of NHLBI	European or African	http://www.ncbi.nlm.nih.gov/pubmed/23690449
*VWF*	rs150077670	VWF antigen	4468	0.0044	–34.5 (12.7)	WES	ESP of NHLBI	European or African	http://www.ncbi.nlm.nih.gov/pubmed/23690449
*MBIP*	rs116909374	TSH (thyrotropin)	15,037	0.043	–0.208 (0.032)	WGS + imputation	UK10K	British	DOI: 10.1038/ncomms6681
*SERPINA1*	rs28929474	Height	49,889	0.019	0.1346 (0.0253)	WGS + imputation	UK10K	British	Tachmazidou et al. (in press)
*TP53BP1*	rs575505283	PLT	121,793	0.014	–0.162 (0.019)	WGS + imputation	UK10K and others	European	https://www.ncbi.nlm.nih.gov/pubmed/27668658
*NPRL3*	rs117747069	MCH	120,851	0.037	–0.176 (0.024)	WGS + imputation	UK10K and others	European	https://www.ncbi.nlm.nih.gov/pubmed/27668658
*CDH13*	rs12051272	Adiponectin	3621	0.009	–1.074 (0.156)	WGS	UK10K	British	http://www.ncbi.nlm.nih.gov/pubmed/26367797
*APOH*	rs1801689	PLT	13,5097	0.033	0.104 (0.012)	WGS + imputation	UK10K and others	European	https://www.ncbi.nlm.nih.gov/pubmed/27668658
*ABCA6*	rs77542162	Cholesterol, total	35,000	0.034	0.14	WGS + imputation	GoNL	Dutch	http://www.ncbi.nlm.nih.gov/pubmed/25751400
*ABCA6*	rs77542162	LDL cholesterol	35,000	0.034	0.135	WGS + imputation	GoNL	Dutch	http://www.ncbi.nlm.nih.gov/pubmed/25751400
*B4GALT6*	rs113107469	FT4 (free thyroxine)	13,649	0.032	0.223 (0.037)	WGS + imputation	UK10K	British	DOI: 10.1038/ncomms6681
*C3*	rs147859257	AMD	52,578	0.0055	3.13 (1.99–4.91)	WGS + imputation	deCODE	Icelandic	http://www.ncbi.nlm.nih.gov/pubmed/24036950
*LDLR*	rs72658867	ApoB	3621	0.012	–0.538 (0.119)	WGS	UK10K	British	http://www.ncbi.nlm.nih.gov/pubmed/26367797
*LDLR*	rs72658867	LDL cholesterol	3621	0.012	–0.584 (0.112)	WGS	UK10K	British	http://www.ncbi.nlm.nih.gov/pubmed/26367797
*APOE*	rs7412	LDL cholesterol	6602	0.036	–0.645 (0.053)	WGS + imputation	SardiNIA	Sardinian	http://www.ncbi.nlm.nih.gov/pubmed/26366554
*APOE*	rs7412	Triglycerides	6602	0.036	–0.544 (0.053)	WGS + imputation	SardiNIA	Sardinian	http://www.ncbi.nlm.nih.gov/pubmed/26366554
*APP*	rs63750847	Alzheimer’s disease	71,743	0.00467	0.189	WGS + imputation	deCODE	Icelandic	http://www.ncbi.nlm.nih.gov/pubmed/22801501
*LGR4*	hg18_chr11:27369242_A	BMD	95,085	0.00174	–0.75 (0.16)	WGS + imputation	deCODE	Icelandic	http://www.ncbi.nlm.nih.gov/pubmed/23644456
*PDX1*	hg18_chr13:27396636delT	Type 2 diabetes	278,254	0.00198	2.47	WGS + imputation	deCODE	Icelandic	http://www.ncbi.nlm.nih.gov/pubmed/24464100

*AF* allele frequency, *AMD* age-related macular degeneration, *BMD*, bone mineral density, *CI* confidence interval, *ESP* Exome Sequencing Project, *FEV* forced expiratory volume, *FVC* forced vital capacity, *HDL* high-density lipoprotein, *LDL* low-density lipoprotein, *MCH* mean cell haemoglobin, *MCV* mean cell volume, *NA* not applicable, *NHLBI* National Heart, Lung, and Blood Institute, *OR* odds ratio, *PLT* platelet count, *SE* standard error, *WES* whole-exome sequencing, *WGS* whole-genome sequencing, *VLDL* very low-density lipoprotein, *VWF* Von Willebrand factor

A study by the UK10K Project exploited low-read-depth WGS and focused on 64 different quantitative cardiometabolic traits in the general UK population [21, 39]. While yielding initial discoveries of rare informative alleles [22, 124–126], these initial efforts have highlighted a clear need to increase the statistical power of studies of complex human disease, particularly to target the contribution of rare variation. Further, they showed that highly penetrant alleles contributing to phenotypic variance of cardiometabolic traits are likely to be found at frequencies well below 1% in the general European population, but are poorly tagged by imputation reference panels, suggesting that direct assessment through genome sequencing will be required to comprehensively access this frequency range for complex traits.

deCODE gathered genotypic and medical data of more than half of the Icelandic population [127]. They generated a population-specific reference imputation panel based on WGS data for approximately 2000 study participants. They then applied imputation not only to the approximately 90,000 participants with genome-wide SNP arrays available, but also to over 250,000 participants where genotypes could be inferred from comprehensive genealogical records; this led to novel discoveries for a range of different complex traits and diseases. As one example, Styrkarsdottir et al. [128] identified a nonsense variant in *LGR4* associated with low bone mineral density (osteoporosis). The study included 4931 individuals with low bone mineral density and 69,034 individuals as control group. Steinthorsdottir et al. [129] discovered four previously unreported rare and low-frequency variants in *CCND2*, *PAM* and *PDX1* genes affecting risk of T2D. Helgason et al. [130] found a rare variant in the *C3* gene associated with age-related macular degeneration. Also, rare variants in *TREM2* and *APP* genes were associated with Alzheimer’s disease [131, 132]. Further, this project identified 6795 autosomal LoF SNPs and INDELs in 4924 genes of which 7.7% were homozygotes or compound heterozygotes with a MAF below 2% [122], boosting further effort to study gene inactivation in humans. Recently, a rare variant in *ASGR1* gene was found to lower the risk of a heart attack by more than one-third in Icelanders [133]. The function of this gene needs still to be elucidated, but possibly it could be protective against heart disease with an alternative mechanism rather than acting on blood lipids, making it a potentially promising drug target to prevent heart disease.

The Genome of the Netherland (GoNL) project used WGS to characterise DNA sequence variation in the Dutch population, focusing on a representative sample consisting of 250 trio-families from all provinces in the Netherlands [41, 134]. Significant improvement in the imputation quality for rare variants (MAF 0.05–0.5%) compared with the 1000GP were demonstrated for the Dutch population, illustrating the value of using large, population-specific reference panels for imputing rare variants [135]. Further, use of this panel led to the identification of a rare deleterious missense variant in *ABCA6* associated with LDL-C and TC in the Dutch population [136].

Similarly, the African Genome Variation Project, consisting of dense genotypes from 1481 individuals and whole-genome sequences from 320 individuals across sub-Saharan Africa, demonstrates the importance of adding population specific cohorts to existing reference panels to improve imputation accuracy [137] to account for the greater genetic diversity in these regions compared with the other populations who have expanded out of Africa.

The SardiNIA project is a longitudinal study including genetic and phenotypic data for 1257 multigenerational families from four villages in the Lanusei valley in Sardinia, Italy. In a recent study, WGS was performed in a total of 2120 participants [43], discovering 76,000 variants that were common in the SardiNIA study (frequency >5%) but rare elsewhere (<0.5% in the 1000GP). This study identified 14 associations for lipid levels (including two major new loci) and 19 for inflammatory markers (including two novel loci). In a companion study [138], the authors also identified five variants regulating haemoglobin levels at previously undetected loci (*MPHOSPH9*, *PLTP-PCIF1*, *ZFPM1* (*FOG1*), *NFIX* and *CCND3*), highlighting the importance of sequencing isolated populations in finding variants that may be very rare and possibly not present in other populations.

The Cohorts for Heart and Aging Research in Genomic Epidemiology Consortium (CHARGE) design includes five prospective cohort studies from the USA and Europe: the Age, Gene/Environment Susceptibility—Reykjavik Study, the Atherosclerosis Risk in Communities Study, the Cardiovascular Health Study, the Framingham Heart Study and the Rotterdam Study [139]. Among the studies published by this project (Table 2), one for instance identified rare variants with large effects associated with HDL-C levels through WGS of individuals sampled from the tails of the phenotypic distribution, some of which overlap with previously identified variants in Mendelian disorders [140].

ENGAGE was a successful consortium effort bringing together data from large-scale research in genetic and genomic epidemiology from population cohorts to be translated into information relevant for future clinical applications [141]. In a recent study based on imputation using the 1000GP, 15 loci with low-frequency and ten loci with missense lead-SNPs and two loci with an accumulation of rare variants were found to be associated with lipid levels, and were also found to increase the proportion of variance explained for LDL-C and TC [142].

As part of the National Heart, Lung, and Blood Institute (NHLBI) Exome Sequencing Project, Emond et al. [105] identified missense variants in *DCTN4* that are associated with resistance to *Pseudomonas aeruginosa* infections. This study was conducted using an extreme phenotype design in which WES was carried out on patients with cystic fibrosis (*n* = 91). A large WES study (*n* = 2005), also part of the Exome Sequencing Project, identified a novel gene, *PNPL5*, affecting LDL-C levels [143]. Do et al. [144] found rare variants in *LDLR* and *APOA5*, increasing risk for myocardial infarction. In another study, rare and common variants were found to be associated with von Willebrand disease and factor VIII levels in African Americans [145]. Finally, analysis of whole exome sequences of 3734 participants of European or African ancestry identified rare mutations disrupting *APOC3* function associated with lower levels of plasma triglycerides and a reduced risk of coronary heart disease for carriers of these mutations [104].

A large-scale sequencing study by the GoT2D and T2D-GENES consortia [19] investigated lower frequency variants discovered from WGS of 2657 European individuals with and without T2D and WES of 12,940 individuals from five ancestry groups. The variants discovered were not sufficient to explain the large fraction of heritability missed from previous GWASs.

Extending to neuropsychiatric disorders, a recent study identified rare LoF variants in the *SETD1A* gene to be associated with schizophrenia. The WES study of 4264 schizophrenia cases, 9343 controls and 1077 trios identified three de novo mutations and seven LoF variants found in cases in the discovery cohort but none in controls. Two analytical approaches, one based on Fisher’s method to combine de novo and case–control *p* values and the other using the transmission and de novo association (TADA) model, were used in the study [146].

Finally, cancer such as breast cancer has a high incidence worldwide with 5–10% of cases associated with highly penetrant germline susceptibility alleles. *BRCA1* and *BRCA2* are the first genes found to be associated with a higher predisposition to breast cancer [147]. Most *BRCA1* and *BRCA2* pathogenic variants are predicted to produce a truncated protein product and thus loss of protein function [148]. However, the prevalence of *BRCA1* and *BRCA2* mutations is only approximately 24% [149, 150]. Recently, exome sequencing has uncovered substantial locus heterogeneity among affected families without *BRCA1* or *BRCA2* mutations [151, 152]. The new pathogenic variants are rare, posing challenges to estimation of risk attribution through patient cohorts. Among these, rare monoallelic LoF variations within the *PALB2* gene (partner and localiser of *BRCA2*) are associated with breast cancer at a risk two to four times that among non-mutation carriers [153].

These and other examples illustrate the value of different designs, including sequencing population-specific cohorts to enhance the imputation quality of rare and low-frequency variants, exploiting population isolates, and sequencing of extremes of phenotypic traits. Despite limitations of power and resolution, rare variant association studies are becoming increasingly mature. The majority of associations with low-frequency and rare variants demonstrate relatively small effects on complex traits and disease. Interestingly, a study conducted by Wood et al. [154] in an Italian cohort (InCHIANTI) specifically compared phenotypic effects of low-frequency and rare variants to those of common variants. While some low-frequency variants with larger effect sizes (and similarly phenotypic variance explained) were detected, these represented a very small proportion of all association. This suggests that, particularly for outbred populations, greater sample sizes will be necessary to realise the potential of RVASs to identifying new genes involved in human disease pathways and biology.

## Future prospects

Despite the success of GWASs in identifying thousands of robust associations with complex diseases and traits, few examples of these results have been successfully translated into clinical use [118, 155, 156]. Nevertheless, GWAS loci have been shown to increase the therapeutic validity of selected targets by twofold compared with previous target selection [157]. Substantial decreases in sequencing costs, coupled with increases in throughput afforded by massively parallel sequencing, offer the promise to greatly boost the discovery of highly informative rare and low-frequency genetic variants through WES and WGS. Advances in phenotyping (including multivariate measures of traditional disease risk factors, disease-relevant endpoints derived from electronic health records or molecular traits driven by advances in functional and cellular genomics) will further boost the power of these genomic approaches. Multiple areas of research will benefit from these enhancements. First, they will lead to discoveries of highly informative rare alleles, including LoF mutations, associated with risk of disease. Second, they will provide more powerful genetic tools to assess the causal contribution of novel biological pathways to disease risk through Mendelian randomisation approaches. Finally, they will enable efforts to dissect and refine understanding of causal regulatory variants through genome-scale molecular and cellular assays. Thus, the discovery of associations driven by low-frequency and rare variants are expected to contribute to efforts to validate therapeutic targets, for instance by identifying alleles that mimic the effect of modulating drug target genes, which can inform the likelihood of success in treating disease by modulating biological pathways through novel and existing drugs. These approaches thus offer great promise for reducing the attrition rate in drug development by identifying new drugs with higher efficacy and by informing repositioning of existing drugs towards new disease indications.

## Additional file

Additional file 1: All rare variants (allele frequency <5%) that were discovered across different traits, together with chromosomal position (GRCh37), mapped gene, disease or traits, sample size, allele frequency, effect size, population and reference. (XLSX 39 kb)

## Abbreviations

CAST: Cohort allelic sums test
CMC: Combined multivariate and collapsing
GWAS: Genome-wide association study
INDEL: Insertion-deletion
MAF: Minor allele frequency
RVAS: Rare variant association study
SKAT: Sequence kernel association test
SNP: Single-nucleotide polymorphism
SNV: Single-nucleotide variant
T2D: Type 2 diabetes
WES: Whole-exome sequencing
WGS: Whole-genome sequencing
